# Curative Electricity

**Published:** 1883-12

**Authors:** 


### CURATIVE ELECTRICITY.

### THE KIDDER APPARATUS STILL THE BEST IN THE WORLD.

The large extent to which electricity is now utilized in various departments of medical treatment has been the means of inducing a good many concerns that are not properly equipped for such work to go into the business of making electrical instruments. Such concerns turn out apparatus that is really not worth using and that causes nothing but disappointment where it is used. It is important for those who want to get the very best results to stand by the old reliable system invented by DR. JEROME KIDDER and now controlled by the Jerome Kidder Manufacturing Company of 820 Broadway. The late Dr. Jerome Kidder was probably the ablest man who ever gave attention to modern electro-medical science, and his apparatus have certainly never been equaled. They have won the chief honors wherever they have been exhibited, notably on the following occasions:

The highest award at Centennial Exhibition, 1876, highest award at the American Institute, of New York, from 1872 to 1883 inclusive, and in 1875 the gold medal for the best electro-magnetic machine, either in this country or abroad. Also four silver medals, highest award given at Cincinnati Industrial Exposition of 1881 and 1882; also silver medal, fall 1883. Silver medal awarded at Charleston, S. C., Exhibition of 1882. Also the highest medal at the Exhibition just closed at Louisville. Prof. Ogden Doremus and other high scientific authorities have declared in the strongest terms their opinion that the Kidder batteries are unequaled for all curative purposes—and in fact there cannot be any justly founded contrary opinion.

